# High endothelial venules are rare in colorectal cancers but accumulate in extra-tumoral areas with disease progression

**DOI:** 10.4161/2162402X.2014.974374

**Published:** 2015-04-02

**Authors:** Diana Costa Bento, Emma Jones, Syed Junaid, Justyna Tull, Geraint T Williams, Andrew Godkin, Ann Ager, Awen Gallimore

**Affiliations:** 1 Infection and Immunity; School of Medicine; Henry Wellcome Building; Cardiff University; Cardiff, UK; 2 Institute of Medical Genetics; University Hospital of Wales; Cardiff, UK; 3 Institute of Cancer and Genetics; Cardiff University School of Medicine; Cardiff, UK

**Keywords:** colorectal cancer, high endothelial venules, lymphoid aggregates/follicles, T cells, tumor infiltrating lymphocytes and tertiary lymphoid organs, A/F, aggregate/ follicle, CRC, colorectal cancer, HEV, high endothelial venule, TILs, tumor-infiltrating lymphocytes, Treg, regulatory T cell.

## Abstract

Prolonged patient survival after surgical resection, is associated with a higher cytotoxic and memory T cell density within colorectal cancers (CRC). High endothelial venules (HEVs) are specialized blood vessels present in secondary lymphoid organs (SLO) that allow ingress of naïve and central memory T cells from the blood. It has been proposed that HEVs in tumors might serve as a similar route of entry for lymphocytes into the tumor and result in an improved prognosis. The present study aimed to characterize HEVs and their microenvironment in resected tumors from colorectal cancer patients (*n* = 62). We observed HEVs in association with lymphoid aggregates in 49 out of 62 patients. However, these HEV^+^ lymphoid aggregates were largely at the invasive margin of the tumor and although there was an association with lymphocytes and HEVs at the invasive margin (*p* = 0.002) there was only a very weak association with tumor infiltrating lymphocytes. Indeed, lymphoid aggregates were associated with more advanced disease (Dukes’ stage C) and did not indicate a favorable prognosis.

## Introduction

CRC causes 600,000 deaths annually with 1 million new cases emerging every year in the Western world.^1^ Primary tumors are often removed by surgery, but it remains the case that 40–50% of patients experience tumor relapse and die of CRC.^2^ Colectomy specimens are histologically analyzed and the CRC staged according to an international (TNM) or Dukes’ classification (Dukes’ stages A–C).^3^ Patients with tumors confined to the bowel wall (Dukes’ A) have a > 90% chance of cure while fewer than half of those with local lymph node metastases (Dukes’ C) survive more than 5 years post-resection.^4^ The extent of T cell infiltration acts as an independent prognostic factor to histopathological staging^5-7^; thus, it is likely that tumor-infiltrating T cells may not only play a critical role in limiting disease progression but also reflect a population of antitumor T cells which can control disease recurrence after surgical resection.

HEVs are specialized blood vessels usually present in the paracortex and interfollicular areas of SLO which allow migration of L-selectin^+^ cells (e.g. naïve and central memory T cells)^8^ from the blood into the lymphoid organ.^9^ HEV comprise cuboidal endothelial cells and express on their surface peripheral node addressins (PNAds, detected via the well characterized MECA-79 antibody)^10^ which act as ligands for L-selectin binding. Also, the presence of dendritic cells appears to be crucial for the maintenance of the HEV phenotype.^11^ Neogenesis of HEVs has been reported under conditions of chronic inflammation often in association with *de novo* formation of tertiary lymphoid organs (TLO) comprising a cellular network of B and T cells.^9,12^ Recently, the presence of HEVs in lymphocytic rich areas has been reported in breast, lung, colon, and ovarian carcinomas and in melanomas.^13–16^ According to the more detailed studies of breast cancer and melanoma, elevated HEV numbers in the tumor correlates with a better patient outcome and a longer tumor and metastasis-free survival,^13,14^ leading the authors to propose that HEV neogenesis facilitates entry of effector antitumor lymphocytes into the tumor site. In addition, we have previously shown in mice that control of methylcholanthrene (MCA)-induced tumors after regulatory T cell (Treg) depletion was dependent on high densities of T cells within the tumor microenvironment and was associated with the presence of HEVs.^17^

We have previously examined in detail the phenotype of TILs in CRCs derived from patients with various stages (Dukes’ A–C) of disease.^18–20^ However, although the lymphocyte cellularity of tumors varies markedly, the mechanism of this remains unclear. On the basis of our murine studies^17^ and preliminary data relating to TILs in CRC, we re-examined tissue sections to ascertain whether HEVs were present and associated with TIL density. Specifically, the study was designed to assess whether the presence or absence of HEVs was associated with (i) the formation of lymphoid structures at the site of the CRC, (ii) the number of T cells present within the tumor mass, (iii) the clinical stage of disease, and (iv) clinical outcome.

## Results

### Human colorectal cancers are associated with development of extra-tumoral but rarely intra-tumoral HEVs

**Figure 1.**
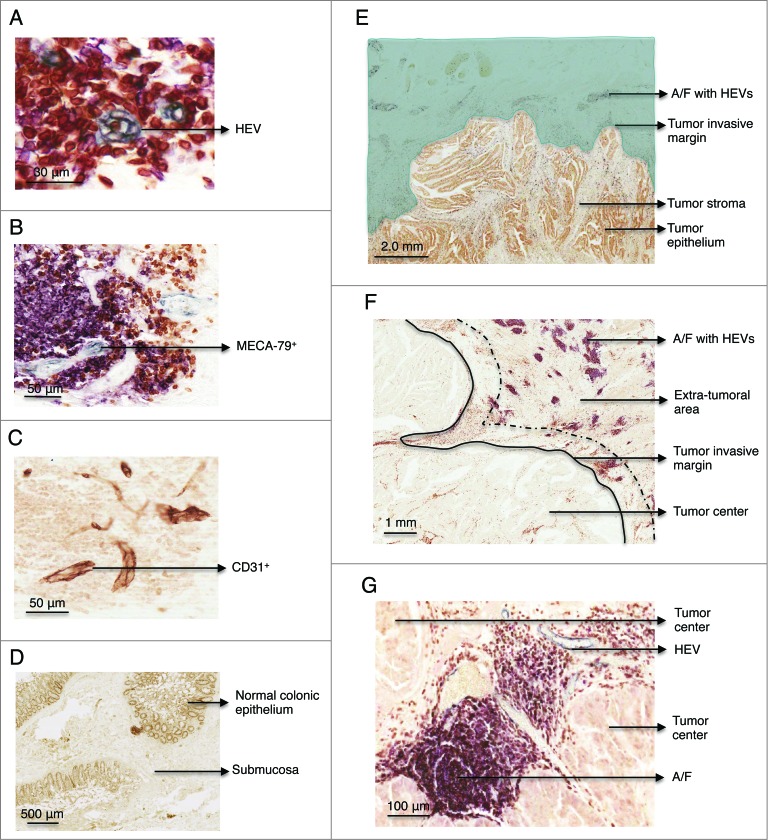
Immunohistochemistry staining of MECA-79^+^ and CD31^+^ vessels, CD3^+^, CD20^+^ cells in paraffin-embedded formalin fixed samples of healthy colon and colorectal tumor. (**A**) Detail of a HEV in gray (MECA-79+). Consecutive sections stained with (**B**) MECA-79 (gray), CD3 (brown), CD20 (pink) and (**C**) CD-31. (**D**) Representative example of a healthy colon lacking lymphoid A/F. The section was stained with anti-CD3, CD20 and MECA-79. (**E**) Overview of a colorectal adenocarcinoma. The area highlighted in green illustrates the extra-tumoral area, surrounding the tumor but not the tumor itself. HEVs were located in the extra-tumoral area at the advancing tumor invasive margin. (**F**) Lymphoid aggregates were located ahead of the tumor edge/tumor invasive margin. The area between the black line and the dashed line indicates where T cells were enumerated. The distance between these two lines is equivalent to one high power field at 600x magnification. (**G**) Detail of a lymphoid aggregate/follicle with a HEV within the tumor center. HEV, high endothelial venule. A/F, lymphoid aggregates/ follicles.

We first sought to determine whether HEVs are present in normal and/or malignant colon. HEVs were identified through their characteristic cuboidal and plump morphology, MECA-79 staining, and co-expression of the pan-endothelial cell marker, CD31 (**Figs. 1a–c**). No HEVs were detected in normal colon other than in the context of gut-associated lymphoid tissue (GALT) (**Fig. 1d** and **Fig. S1**). Striking differences were observed in CRCs where HEVs were often observed in the extra-tumoral area, positioned ahead of the tumor invasive margin (**Figs. 1e and f**). In this extra-tumoral area, highlighted in green in **Fig. 1e**, HEVs were always found in association with a concentration of CD3^+^ T and CD20^+^ B cells (**Fig. 1f**). HEVs were identified in the extra-tumoral area of 49 out of 62 patients. Staining with isotype control antibodies is shown in **Fig. S1**.

In contrast to previous reports of breast cancer^13^ and melanoma,^14^ HEVs were rarely observed within CRC tumor stroma or epithelium (tumor center). In the small number of tumors where HEVs were detected within the tumor center (*n* = 8), most were atypical due to the flat morphology of the cells (**Fig. 1g**) and in some cases absence of lymphocyte aggregates (**Fig. S2**).

**Figure 2.**
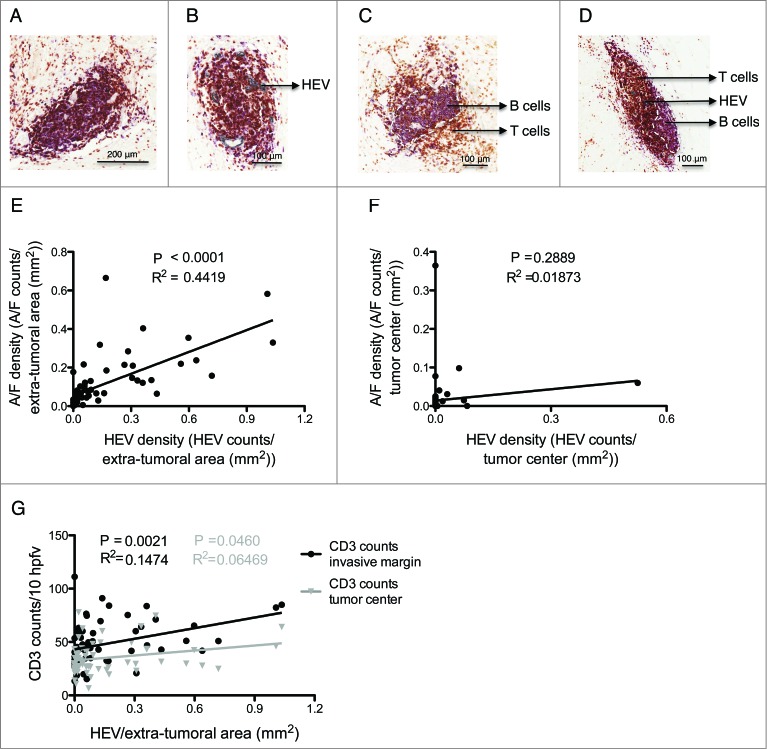
Relationship between lymphoid aggregates, high endothelial venules and T cell infiltration at the invasive margin and the tumor center. (**A**) Lymphoid aggregate without HEVs. (**B**) Lymphoid aggregate with HEVs. (**C**) Lymphoid follicle without HEVs. (**D**) Lymphoid follicle with HEVs. (**E**) Lymphoid A/F density vs. HEV density in the extra-tumoral area of all tumors (*n* = 62). (**F**) Lymphoid A/F density vs. HEV density in the tumor center. (**G**) HEV density in the extra-tumoral area vs. CD3 counts at the tumor invasive margin (black) and in the tumor center (gray) (*n* = 62). HEV, high endothelial venule. A/F, lymphoid aggregate/ follicle. HPFV, high power field of view. The white dashed lines in (**C**) and (**D**) represents organized B cell areas in follicles. *p* < 0.05 was considered significant.

As reported by others,^21^ in some cases, the MECA-79 antibody also stained the tumor epithelium. This was not indicative of HEV, as the morphology of the stained cells was clearly not HEV-like and these structures did not co-express the pan-endothelial marker CD31 indicative of a vessel (data not shown).

### HEVs are associated with lymphoid aggregates and lymphocyte numbers at the tumor invasive margin

HEV-associated lymphocyte clusters observed in the extra-tumoral area ranged from relatively disorganized lymphoid aggregates comprising collections of T and B cells to organized follicle-like structures comprising distinct B and T cell areas (**Figs. 2a–d**). Lymphoid aggregates represented the majority of lymphocyte clusters while lymphoid follicles were rarely observed (3.4%). Overall, there was a highly significant correlation between these lymphoid structures and HEV density in the extra-tumoral areas (**Fig. 2e**: *p* < 0.0001, R^2^ = 0.442). The occurrence of HEVs within the tumor center (tumor epithelium and stroma) was extremely low (8 out of 62 samples studied), and when the HEVs were present, there was no significant association with lymphoid aggregates (**Fig. 2f**). While all extra-tumoral HEVs were found in association with lymphocyte aggregates, the reciprocal was not the case (data not shown). The inability to detect HEVs in every lymphocyte aggregate may reflect the 2-dimensional nature of the immunohistochemical analyses rather than be indicative of aggregates lacking HEVs.

Despite the paucity of HEVs within the tumor itself, a key question was whether the presence of HEVs and lymphoid aggregates surrounding the tumor reflected an increase in the TIL number. To determine the impact of these HEVs, total CD3^+^ T cells were enumerated within the tumor center and the tumor margin. A significant association was observed between extra-tumoral HEV density and T cells located within the tumor invasive margin (*p* = 0.002, R^2^ = 0.148) but this association was very weak for the tumor center (*p* = 0.05, R^2^ = 0.065) (**Fig. 2g**).

15% of all CRC are microsatellite instable (MSI)^22^ and such tumors are often infiltrated with high numbers of T cells.^23^ We therefore examined samples with the highest extra-tumoral HEV density, including those with HEVs within the tumor center, and found these to be microsatellite stable (**Table S1**). To confirm that MSI does not drive neogenesis of HEV, four MSI^+^ tumors were subsequently analyzed. No striking differences in HEV density, distinguishing MSI^+^ and MSI^−^ tumors, emerged from this analysis (data not shown).

### Lymphoid aggregates are associated with advanced disease

**Figure 3.**
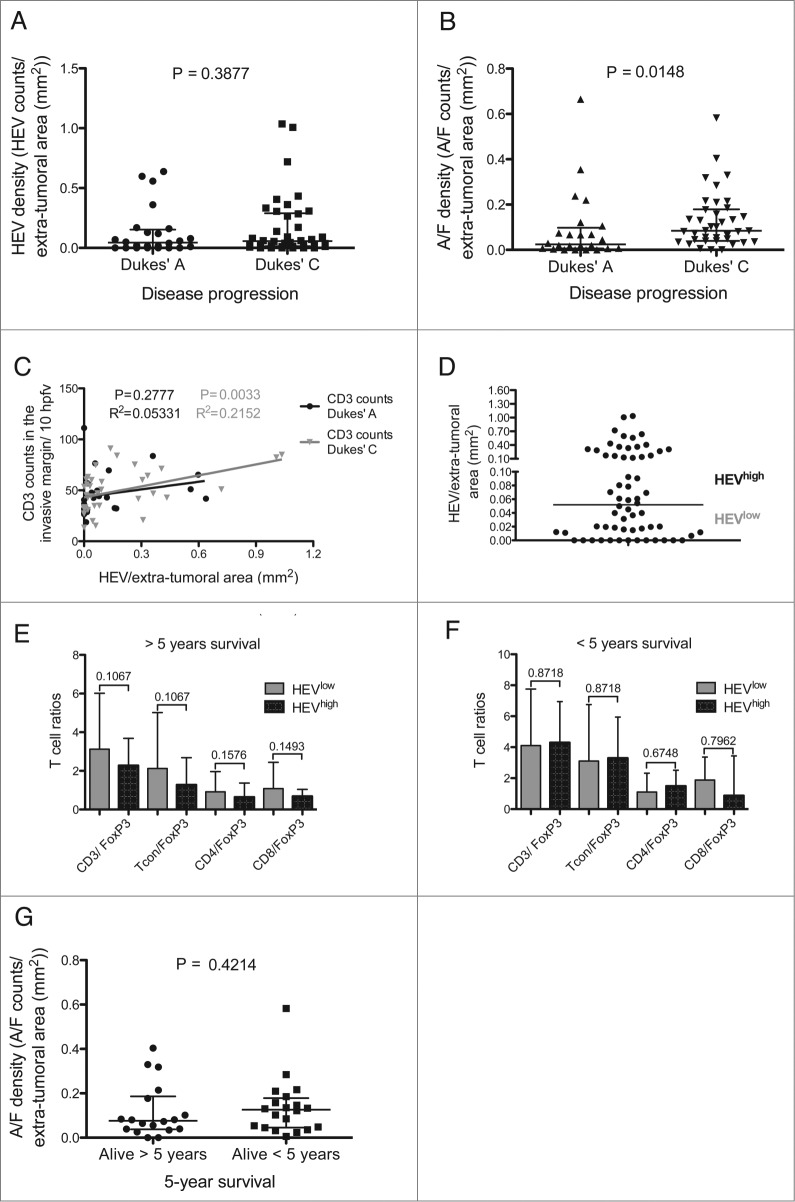
Lymphoid aggregate/follicle density is a marker of disease progression but not survival. (**A**) HEV density in the extra-tumoral area in Dukes’ A and C cases. (**B**) Density of lymphoid aggregates/follicles in the extra-tumoral area in Dukes’ A and C cases. (**C**) CD3 counts per 10 HPFV at the tumor invasive margin vs. HEV density in the extra-tumoral area for Dukes’ A (black) and C (gray). (**D**) The median density in the extra-tumoral area was used to categorize cases into HEV low and HEV high. T cell ratios at the advancing margin of HEV low and high tumors in: (**E**) patients who survived 5 yeras post-colectomy and (**F**) patients that survived less than 5 yeras post-surgery. Conventional T cells (Tcon) were considered to be CD4^+^ and CD8^+^ T cells combined excluding FoxP3^+^ T cells. (**G**) Lymphoid aggregate/follicle density in the extra-tumoral area for Dukes’ C patients who survived for more or less than 5 yeras post-colectomy. The median value with the interquartile range is shown for (**A**), (**B**), (**E**), (**F**) and (**G**). HEV, high endothelial venule, A/F, lymphoid aggregates/follicles. Hpfv, high power field of view. *p* < 0.05 was considered significant.

We next sought to determine whether there was a relationship between the presence of HEVs and/or lymphoid aggregates (present within the extra-tumoral area) and disease progression. As shown in **Fig. 3a**, there was no significant difference between HEV densities and whether a subject had early disease (Dukes’ A: tumor confined to the wall of the bowel) or more advanced disease (Dukes’ C: spread to adjacent lymph nodes). However, the presence of lymphoid aggregates/follicles was significantly associated with more advanced Dukes’ C tumors (**Fig. 3b**: Dukes’ A vs. C, *p* = 0.015. Median number of lymphoid A/ F in Dukes’ A tumors: 0.025, interquartile range 0.006–0.098. Median number of lymphoid A/ F in Dukes’ C tumors: 0.085, interquartile range: 0.039–0.179).

When the relationship between HEVs and T cell infiltration was assessed with respect to disease stage, we found that the association between extra-tumoral HEVs and T cells present at the invasive margin, was only significant in patients with Dukes’ C but not those with Duke's A disease (**Fig. 3c**). To determine whether HEVs were associated with alterations in the composition as well as the number of T cells in the tumor invasive margin, sections were stained with antibodies to CD3, CD8^+^ and FoxP3 and the ratios of CD3^+^, CD4^+^ (CD3^+^CD8^−^) or CD8^+^ T cells to FoxP3^+^ T cells were calculated and assessed with respect to HEV density (**Fig. 3d**). No significant association was observed suggesting that extra-tumoral HEVs play no role in influencing the type of T cells present at the invasive margin in patients that survived 5 years post-colectomy (**Fig. 3e**) or patients that survived less than 5 years (**Fig. 3f**).

### Lymphocytic aggregates are not associated with patient prognosis

Since lymphocytic aggregates were present in the extra-tumoral areas of individuals with advanced disease, we determined whether these were associated with a poor prognosis. Of the Dukes’ A tumors analyzed, disease recurrence occurred in only three subjects, thus the analysis was focused in samples from patients with Dukes’ C tumors. These were divided according to whether the patients had succumbed to disease within 5 years of colectomy or survived > 5 y. No significant difference between the groups was observed when they were compared for presence of HEVs (data not shown) or for numbers of lymphocytic aggregates in the extra-tumoral area (**Fig. 3g**). These data imply that presence or absence of HEV and/or lymphoid aggregates is not associated with prognosis in patients with Dukes’ C tumors.

## Discussion

This study focused on the identification and characterization of HEVs in CRC. We established that HEVs form primarily in the extra-tumoral area and rarely within the tumor stroma/epithelium. Moreover, these vessels associate with T and B cells forming aggregates/follicles in agreement with an active role for the vessels in lymphocyte recruitment.^14,16^ The density of these follicles/aggregates is associated with advanced disease as significantly higher numbers, approximately 3.4X (Dukes’ A median 0.02451, range 0–0.6647; Dukes’ C median 0.08455 range 0–0.5823) were observed in Dukes’ C compared to Dukes’ A tumors. While HEVs are found in the GALT of healthy and diseased colon, the HEVs associated with CRC are found more deeply within the bowel wall. These observations suggest that CRC results in initiation of a local immune response driving *de novo* formation of these structures. The increase in lymphocytic aggregate/follicle density in Dukes’ C tumors may be indicative of an intensification of the local immune response corresponding with tumor progression.

The question that arises from these data is whether HEV and/or lymphoid aggregates impacts on patient prognosis. In studies of breast cancer^13^ and melanoma,^14^ high densities of tumor HEVs was associated with an accumulation of T and B cells, high numbers of tumor-infiltrating lymphocytes and a better clinical outcome. We found no relationship however, between lymphoid aggregate densities (and HEVs) with survival of patients with Dukes’ C tumors, implying that while presence of large numbers of these structures reflects advanced disease they are not associated with a favorable prognosis. Indeed, we observed trends for high numbers of lymphoid aggregates in patients who did not survive >5 y post-tumor resection compared to those who did (patients who did not survive >5 post-tumor resection median 0.1260, range 0–0.5823; patients who survived >5 y post-tumor resection median 0.0766 range 0–0.4039) suggesting that the presence of lymphoid aggregates/follicles, albeit within the tumor periphery, do not facilitate an effective antitumor response in Dukes’ C tumors. Actually, the data are compatible with the hypothesis that formation of lymphoid aggregates within the extra-tumoral area reflects an immune response driven by the tumor, which may serve to facilitate cancer progression. Presence of lymphoid aggregates in the large intestine have also been reported in inflammatory bowel diseases^24^ and these patients are at greater risk of developing CRC.^25^ Thus, in contrast to the findings in the cancer studies described above, we have found no positive prognostic value to the presence of CRC-driven lymphoid aggregates or HEVs. However, in comparison to breast cancer and melanoma, in CRC HEVs were rarely observed within the tumor epithelium/stroma. It is possible that HEV neogenesis within CRCs is actively inhibited. We have previously shown in a mouse model of carcinogen-induced fibrosarcomas that HEV formation within these tumors is dependent on Treg depletion.^17^ These data raise the interesting possibility that Tregs or perhaps other immunosuppressive mechanisms present within CRCs act to inhibit HEV formation limiting the extent of lymphocyte infiltration into the tumor.

While only early stage (Dukes’ A) and advanced (Dukes’ C) tumors were included in our study, Di Cario et al. recently reported that lymphoid aggregates were associated with a favorable prognosis for Stage II CRCs, but only for those with no node involvement.^16^ A high density of Crohn's like reaction has also been reported to be associated with less advanced disease and microsatellite instability (MSI).^26^ These data suggest that elevated frequencies of lymphoid aggregates in mismatch repair defective tumors, which are thought to be more immunogenic than microsatellite stable tumors, may be beneficial. Collectively these data raise the interesting possibility that the composition of these lymphoid aggregates, and therefore their prognostic significance, alters with disease progression and according to the inherent immunogenicity of the tumor. With this in mind, studies to evaluate T cell activation signals and T cell signatures within these ectopic structures at different disease stages are warranted.

Collectively, the findings of our study indicate key differences in the relationship between ectopic lymphoid structures developing in response to CRC as opposed to those reported in non-small cell lung^27^ and breast cancer.^13^ In the case of the latter tumor, HEVs and a concentration of T and B cells were found within the tumor stroma where their density correlated with the extent of T cell infiltration. In our study, HEVs were rarely found within CRC tumors’ stroma/epithelium and we found no relationship between HEVs in these areas and the extent of T cell infiltration at either of the cancer stages examined. These data suggest that in CRC, intra-tumoral HEVs play a minor role, if any, in recruiting tumor-infiltrating lymphocytes.

In conclusion, this study of CRC-associated HEV and lymphoid aggregate formation revealed that these structures in fact rarely form in tumors, but accumulate in locations close to the tumor invasive margin. These structures form in association with more advanced tumors, suggesting they are a reaction to progressive tumor invasion.

## Materials and Methods

### Tissue samples

We have previously studied the phenotype of TILs in 62 tumor samples (and adjacent healthy bowel) from patients who have undergone a colectomy for known Dukes’ A and C CRC. Patient's age, gender, tumor location, tumor Dukes’ staging and 5 year survival was also obtained (**Table S2**). Local research ethics committee approval was obtained to perform histological analyses of tumor samples in order to explore the extent to which they are infiltrated with lymphocytes.

### Immunohistochemistry

5 μm thick sections were cut with a microtome (Surgipath), mounted, hydrated in xylene before immersion in descending alcohol concentrations and distilled water. Antigen retrieval was performed when necessary (**Table S3**). Slides were washed in phosphate buffered saline (PBS), immersed in 1% hydrogen peroxide diluted in methanol (1% H_2_O_2_/MeOH) for 10 min and washed with PBS. Unspecific binding was blocked with either 2.5% of normal horse or goat serum (Vector laboratories) added for 30 min. The primary antibody was diluted in 1% bovine serum albumin (BSA) in PBS and incubated overnight at 4°C in a humid chamber (**Table S3**) followed by washing in PBS then incubating with Impress secondary antibody (Vector laboratories) for 30 min. Impress anti-Rabbit, anti-Rat or anti-Mouse was used depending on the species of the primary antibody. Vector® chromagen DAB, SG or VIP was used to visualize antibody complexes. Slides were mounted in DPX and left drying overnight at 65°C. Isotype control antibodies were used at the same concentrations and conditions.

### Cell quantification

CD3^+^, CD4^+^ (CD3^+^CD8^−^), CD8^+^, and FoxP3^+^ labeled cells were enumerated on a NIKON microscope by averaging the cell counts from 10 high power (600x) fields of view within both the tumor mass and by the tumor invasive margin.

Sequential sections were scanned with a scanscope (Aperio technologies) and the HEV and aggregate/ lymphocyte analysis performed with ImageScope viewer software (Aperio). HEV density was calculated either by enumerating all the HEVs within the tumor center and dividing it by the corresponding area (in mm^2^), or by enumerating all the HEVs within the extra-tumoral area and dividing by the respective area in mm^2^ so that two regions of the tumor environment were evaluated. In addition, lymphocyte aggregates/follicles composed of B and T cells were enumerated within the extra-tumoral area and the lymphocyte aggregate/follicle density obtained by dividing the number of aggregates/follicles by the extra-tumoral area in mm^2^. GALT as part of the normal bowel was excluded from the lymphocyte aggregate/follicle enumeration.

### Microsatellite instability analysis

Tumor tissue from samples containing HEVs within the tumor center and from samples with the highest HEV density within the extra-tumoral area (for both Dukes’ A and C) was collected and incubated for 1 h at 57°C in the presence of ATL buffer (QIAGEN) and proteinase K followed by a 1 h incubation at 90°C. The EZ1 DNA Tissue kit (QIAGEN) was used with the EZ1 Advanced XL robot (QIAGEN) according to the manufacturer's instructions to purify DNA. The MSI Analysis System (Promega) was used to identify MSI according to the manufacturer's instructions. Following PCR, the 3730 DNA Analyzer (Applied Biosystems) was used to perform capillary electrophoresis. Peak Scanner™ Software Version 1.0 (Applied Biosystems) was used for analysis. For a sample to be considered MSI at least 2 out of the 5 microsatellite loci studied had to be instable.

### Statistical analyses

Prism 5 (GraphPad) was used to perform all of the statistical analyses. The Mann–Whitney statistical test was used for comparison among groups. The Pearson method was used for correlation analyses and both *p*-value and Pearson correlation coefficients (r^2^) are shown. A *p*-value < 0.05 was considered significant.

## Supplementary Material

974374_Supplementary_Materials.zip
