# Supplementary material for: High endothelial venules are rare in colorectal cancers but accumulate in extra-tumoral areas with disease progression
Source: Oncoimmunology. 2015 Apr 2;4(3):e974374. doi: 10.4161/2162402X.2014.974374 (PMC4404788; doi:10.4161/2162402X.2014.974374)
Supplement: 974374_Supplementary_Materials.zip [file koni-04-e974374-s001.zip › 974374_Figures S1-S2 and Tables S1-S3.pdf]

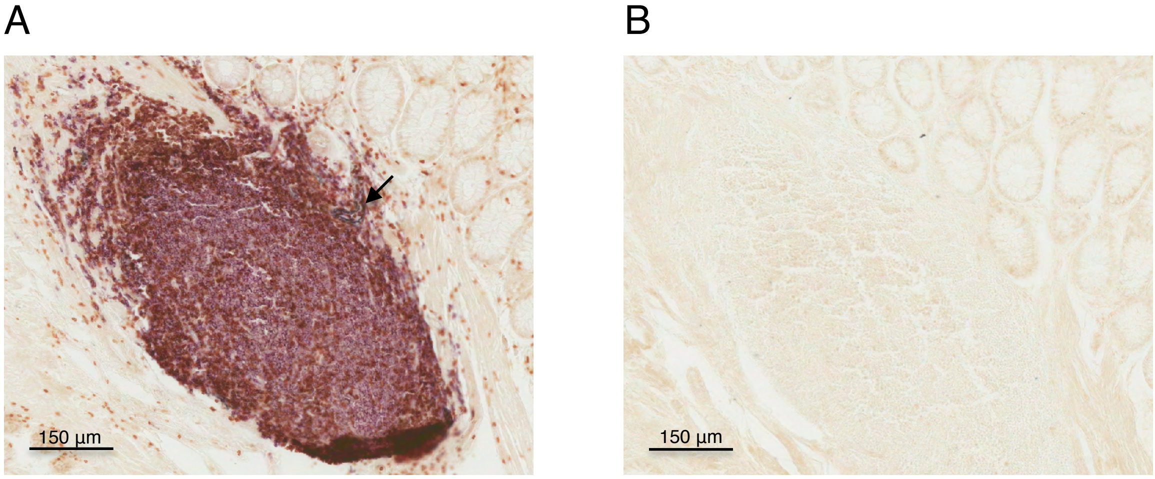

Supplementary figure 1. a) Representative example of a GALT (gut associated lymphoid tissue) stained with CD3 (brown), CD20 (pink) and MECA-79 (grey) showing an HEV (arrow). b) Sequential section stained with Rabbit IgG isotype, mouse IgB2a isotype and Rat IgM isotype.

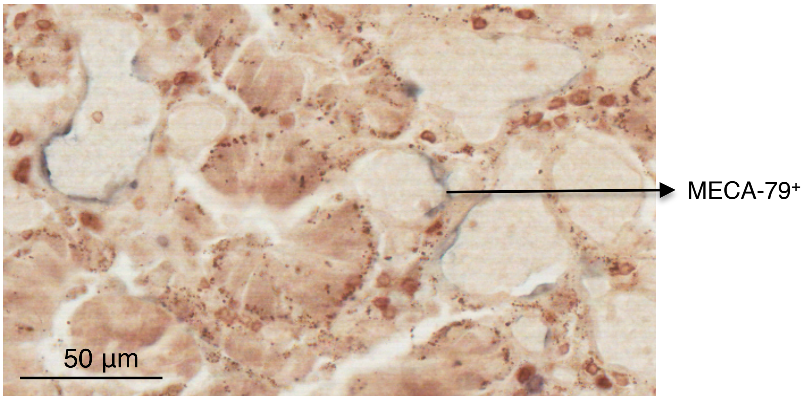

Supplementary figure 2. Example of an atypical HEV devoid of lymphoid tissue present within the tumor centre. Grey indicates MECA-79+ staining.

Supplementary table 1 - Microsatellite status for Dukes' A and C in tumors with the highest density of HEVs in the extra-tumoral area and in tumors with HEVs within the tumor center. Not all the samples tested were usable for MSI testing due to the poor DNA quality. MSS, microsatellite stable.

	Dukes' A	Dukes' C
**Microsatellite status in tumors with the highest HEV density within the extra-tumoral area**	MSS (n=3)	MSS (n=4)
**Microsatellite status in tumors with HEVs within the tumor center**	MSS (n=2)	MSS (n=3)

Supplementary table 2 – Patient details. Information about the patient's age, gender and tumor location was only available for 54, 60 and 53 patients, respectively. (N=62) Columns indicate number and percentage unless otherwise specified. Alive and Dead indicate survival at five years post surgery. SD, standard deviation.

	Number	Percentage
**Age**	Mean 69.4; SD 10.7	
**Sex**		
Male	30	50
Female	30	50
**Tumor location**		
Ascending colon	12	22.6
Transverse colon	2	3.8
Descending colon	2	3.8
Sigmoid colon and rectum	37	69.8
**Dukes' Staging**		
Dukes' A	24	38.7
Alive	21	87.5
Dead	3	12.5
Dukes' C	38	61.3
Alive	18	47.4
Dead	20	52.6
**Five-year survival**		
Alive	39	62.9
Dead	23	37.1

Supplementary table 3 - Antibodies used in immunohistochemistry with the respective concentrations and the antigen retrieval method and buffer applied.

Antigen	Antibody	Concentration (µg/mL)	Species	Antigen retrieval method
CD3	CD3 (DAKO)	2	Rabbit	Sections were microwaved for 8 minutes in 10 mmol/L Tris, 1 mmol/ EDTA buffer, pH9
CD8	CD8 (DAKO)	2	Mouse	
FoxP3	FoxP3 (eBioscience)	1	Rat	
CD20	CD20 (DAKO)	0.45	Mouse	
MECA-79	MECA-79 (Santa Cruz Biotechnology)	2	Rat	
CD31	CD31 (Abcam)	2	Rabbit	
