# Supplementary material for: High endothelial venules are rare in colorectal cancers but accumulate in extra-tumoral areas with disease progression
Source: Oncoimmunology. 2015 Apr 2;4(3):e974374. doi: 10.4161/2162402X.2014.974374 (PMC4404788; doi:10.4161/2162402X.2014.974374)
Supplement: 974374_Supplementary_Materials.zip [file koni-04-e974374-s001.zip › 974374_Figures S1-S2 and Tables S1-S3.pptx]

## Slide 1
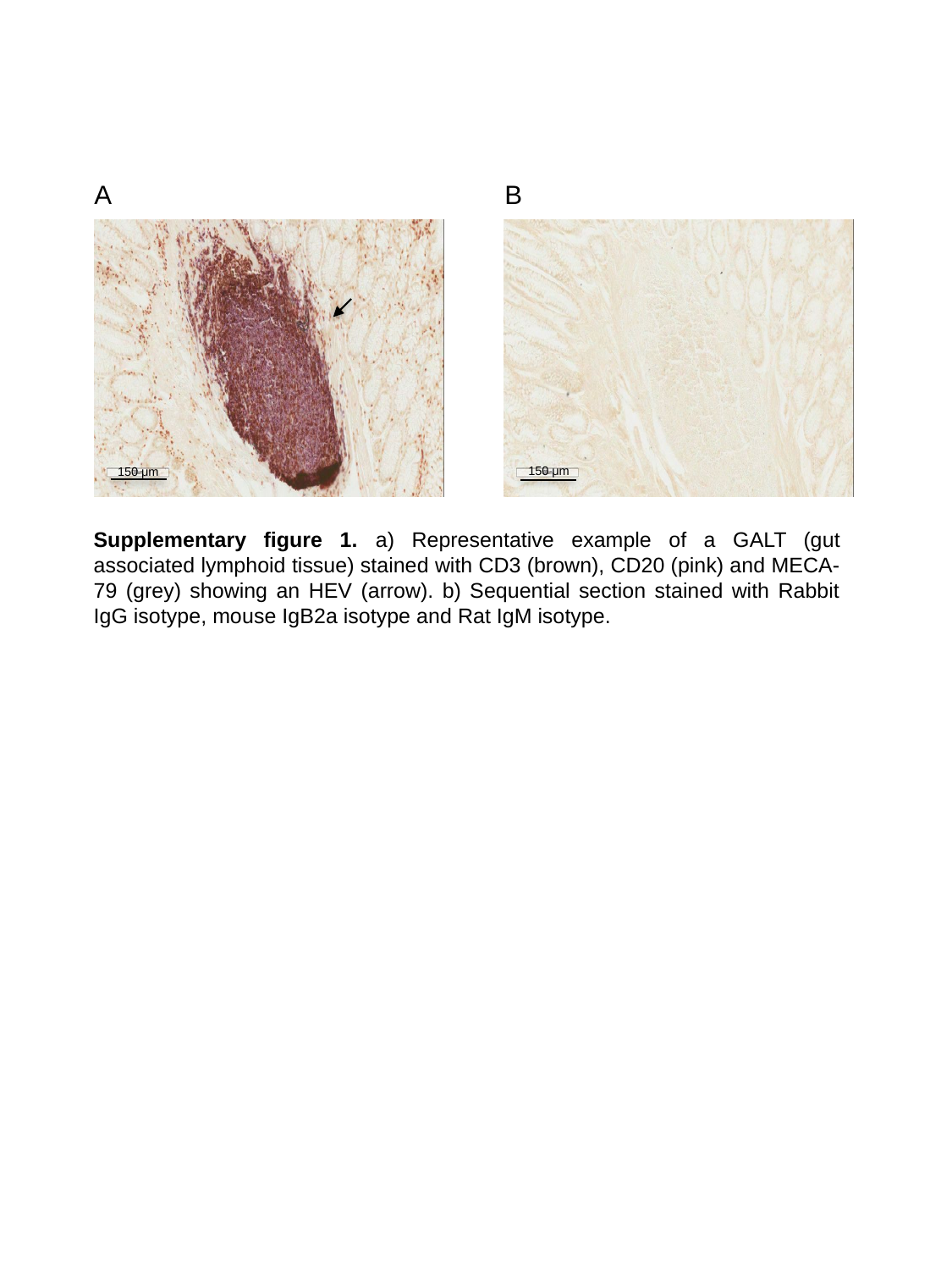

A
B
150 μm
150 μm
Supplementary figure 1. a) Representative example of a GALT (gut associated lymphoid tissue) stained with CD3 (brown), CD20 (pink) and MECA-79 (grey) showing an HEV (arrow). b) Sequential section stained with Rabbit IgG isotype, mouse IgB2a isotype and Rat IgM isotype.

## Slide 2
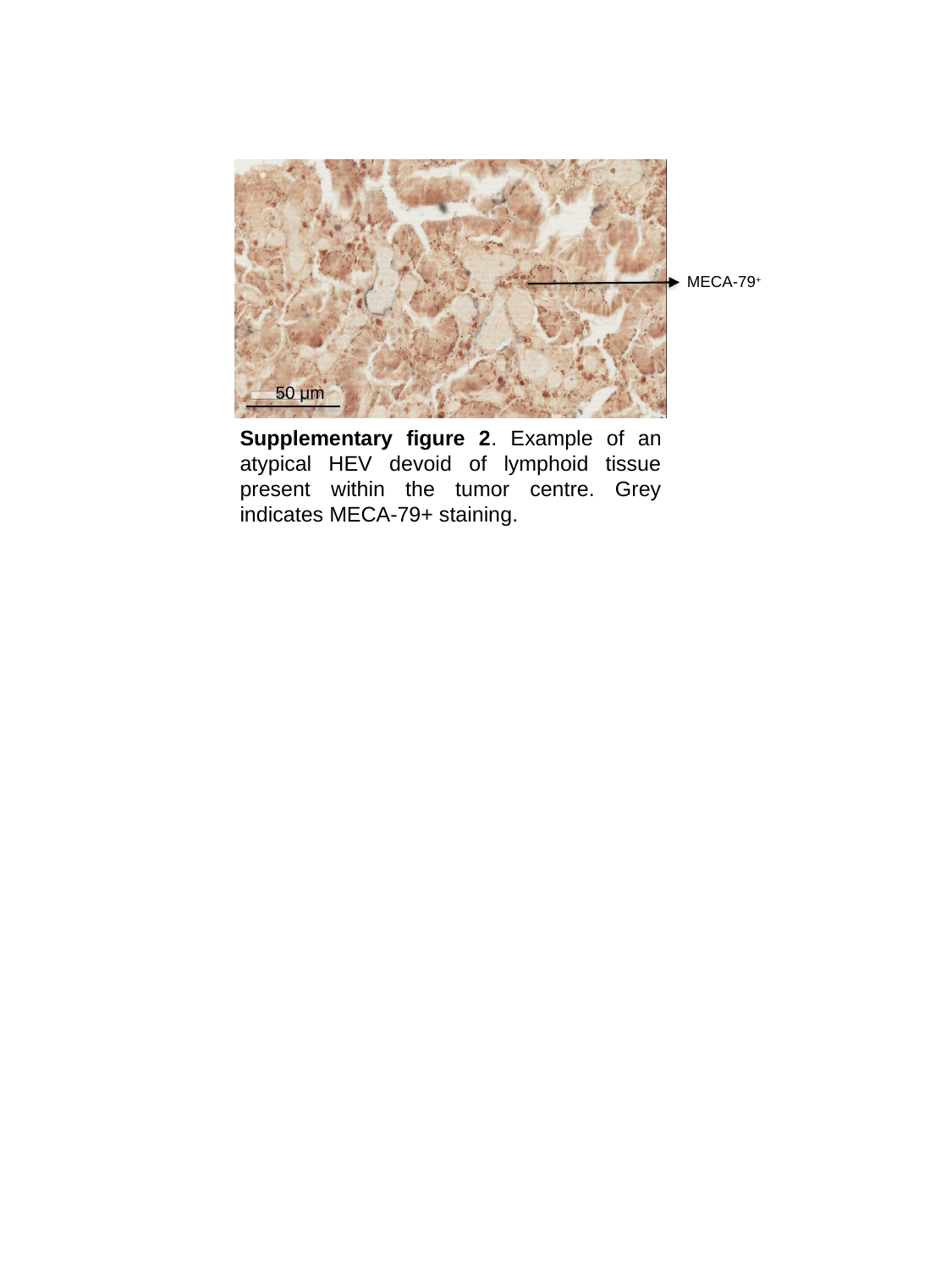

50 μm
MECA-79+
Supplementary figure 2. Example of an atypical HEV devoid of lymphoid tissue present within the tumor centre. Grey indicates MECA-79+ staining.

## Slide 3
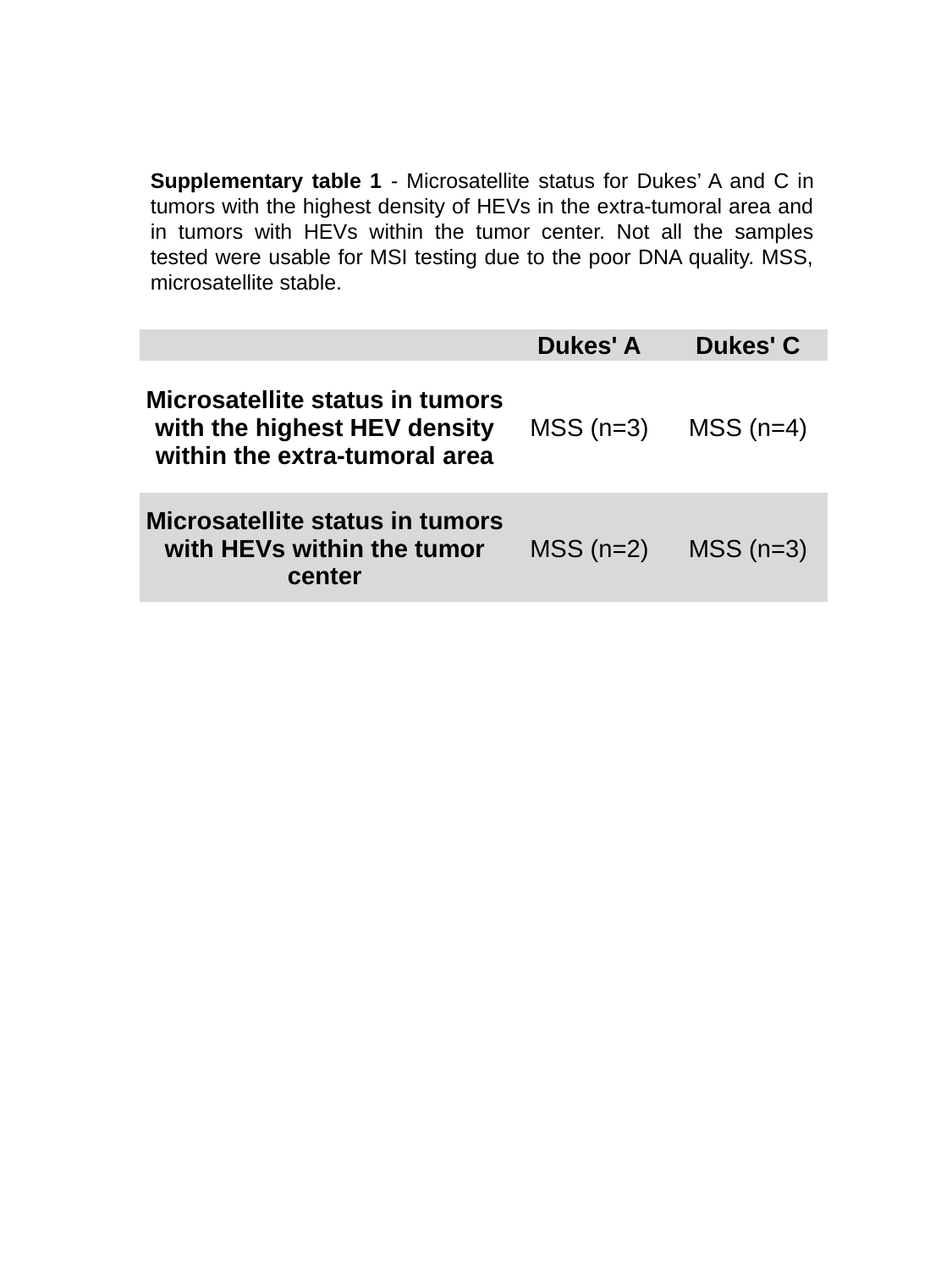

Supplementary table 1 - Microsatellite status for Dukes’ A and C in tumors with the highest density of HEVs in the extra-tumoral area and in tumors with HEVs within the tumor center. Not all the samples tested were usable for MSI testing due to the poor DNA quality. MSS, microsatellite stable.
| | Dukes' A | Dukes' C |
| --- | --- | --- |
| Microsatellite status in tumors with the highest HEV density within the extra-tumoral area | MSS (n=3) | MSS (n=4) |
| Microsatellite status in tumors with HEVs within the tumor center | MSS (n=2) | MSS (n=3) |

## Slide 4
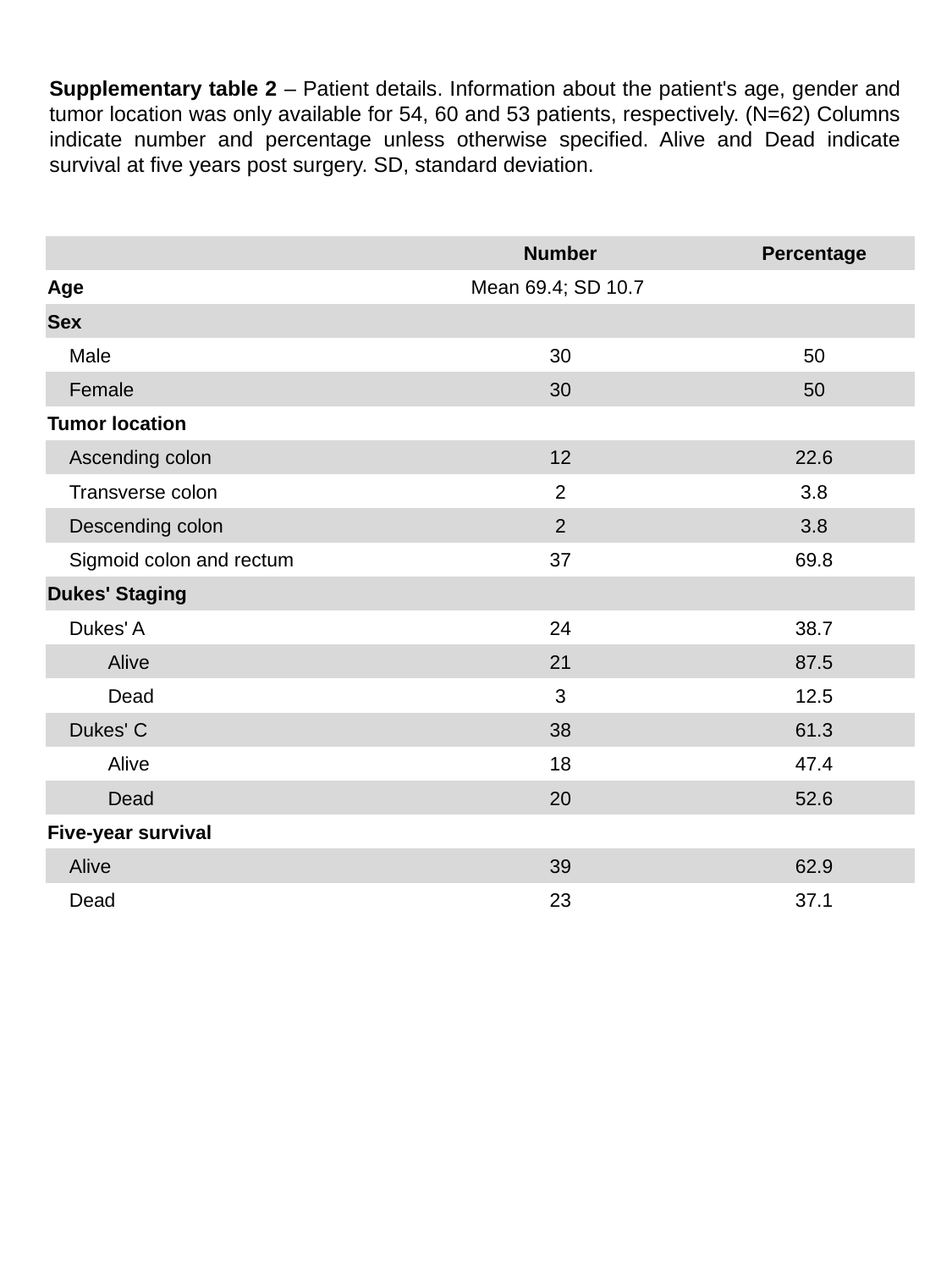

Supplementary table 2 – Patient details. Information about the patient's age, gender and tumor location was only available for 54, 60 and 53 patients, respectively. (N=62) Columns indicate number and percentage unless otherwise specified. Alive and Dead indicate survival at five years post surgery. SD, standard deviation.
| | Number | Percentage |
| --- | --- | --- |
| Age | Mean 69.4; SD 10.7 | |
| Sex | | |
| Male | 30 | 50 |
| Female | 30 | 50 |
| Tumor location | | |
| Ascending colon | 12 | 22.6 |
| Transverse colon | 2 | 3.8 |
| Descending colon | 2 | 3.8 |
| Sigmoid colon and rectum | 37 | 69.8 |
| Dukes' Staging | | |
| Dukes' A | 24 | 38.7 |
| Alive | 21 | 87.5 |
| Dead | 3 | 12.5 |
| Dukes' C | 38 | 61.3 |
| Alive | 18 | 47.4 |
| Dead | 20 | 52.6 |
| Five-year survival | | |
| Alive | 39 | 62.9 |
| Dead | 23 | 37.1 |

## Slide 5
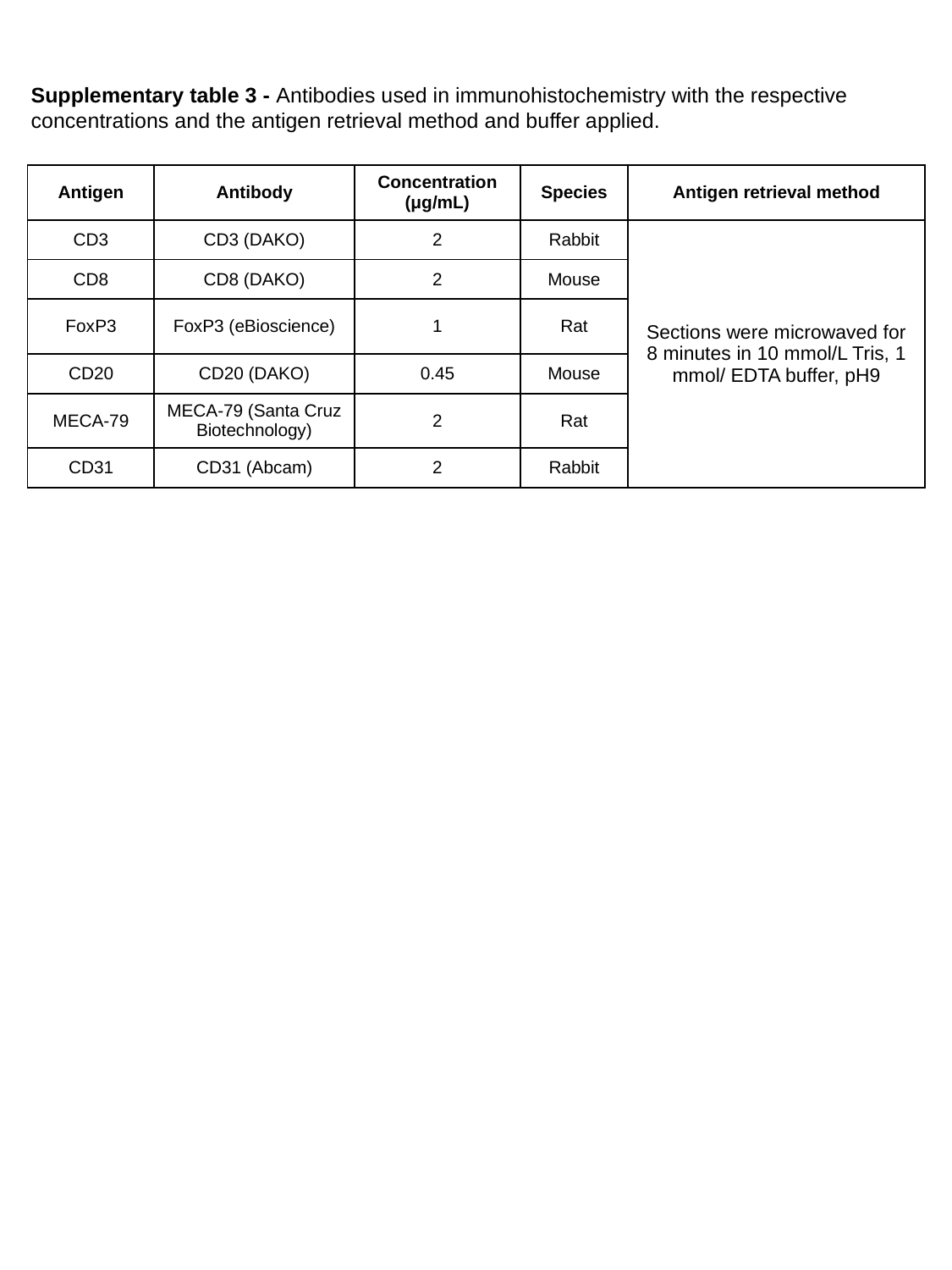

Supplementary table 3 - Antibodies used in immunohistochemistry with the respective concentrations and the antigen retrieval method and buffer applied.
| Antigen | Antibody | Concentration (μg/mL) | Species | Antigen retrieval method |
| --- | --- | --- | --- | --- |
| CD3 | CD3 (DAKO) | 2 | Rabbit | Sections were microwaved for 8 minutes in 10 mmol/L Tris, 1 mmol/ EDTA buffer, pH9 |
| CD8 | CD8 (DAKO) | 2 | Mouse | |
| FoxP3 | FoxP3 (eBioscience) | 1 | Rat | |
| CD20 | CD20 (DAKO) | 0.45 | Mouse | |
| MECA-79 | MECA-79 (Santa Cruz Biotechnology) | 2 | Rat | |
| CD31 | CD31 (Abcam) | 2 | Rabbit | |
