# Supplementary material for: High endothelial venules are rare in colorectal cancers but accumulate in extra-tumoral areas with disease progression
Source: Oncoimmunology. 2015 Apr 2;4(3):e974374. doi: 10.4161/2162402X.2014.974374 (PMC4404788; doi:10.4161/2162402X.2014.974374)
Supplement: 974374_Supplementary_Materials.zip [file koni-04-e974374-s001.zip › 974374_Figures S1-S2.pptx]

## Slide 1
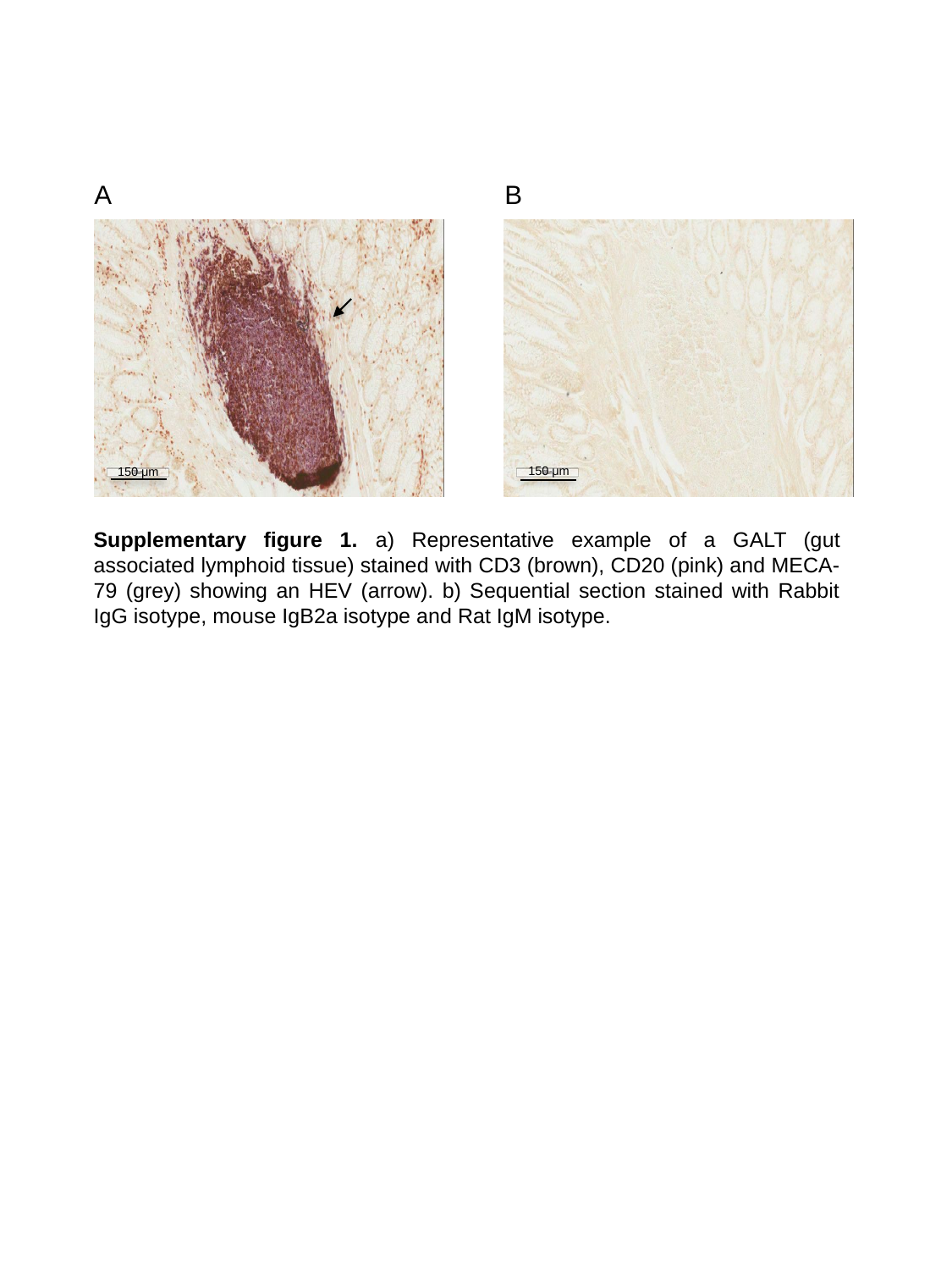

A
B
150 μm
150 μm
Supplementary figure 1. a) Representative example of a GALT (gut associated lymphoid tissue) stained with CD3 (brown), CD20 (pink) and MECA-79 (grey) showing an HEV (arrow). b) Sequential section stained with Rabbit IgG isotype, mouse IgB2a isotype and Rat IgM isotype.

## Slide 2
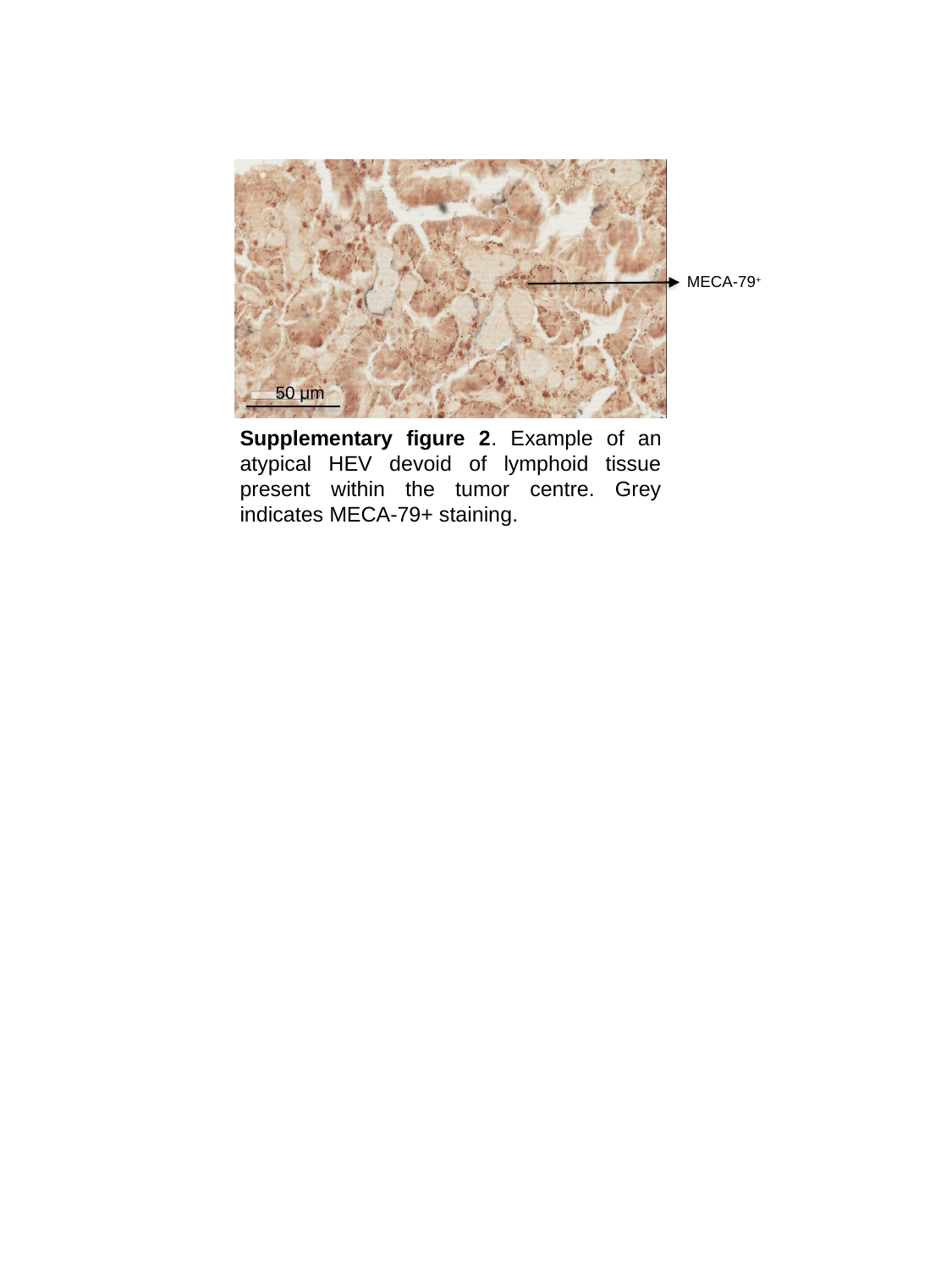

50 μm
MECA-79+
Supplementary figure 2. Example of an atypical HEV devoid of lymphoid tissue present within the tumor centre. Grey indicates MECA-79+ staining.
